# Ethnic minority women’s experiences of accessing antenatal care in high income European countries: a systematic review

**DOI:** 10.1186/s12913-023-09536-y

**Published:** 2023-06-10

**Authors:** Esther Sharma, Pei-Ching Tseng, Angela Harden, Leah Li, Shuby Puthussery

**Affiliations:** 1 Maternal and Child Health Research Centre, Institute for Health Research, University of Bedfordshire, Park Square Rm B201, Luton, Bedfordshire LU1 3JU UK; 2 School of Health Sciences, Division of Health Services Research and Management, City, University of London, Northampton Square London, EC1V 0HB UK; 3 Population, Policy and Practice Programme, Great Ormond Street Institute of Child Health, University College London, London, WC1N 1EH UK

**Keywords:** Antenatal care, Maternal health, Ethnic minority, Europe, Qualitative evidence synthesis

## Abstract

**Background:**

Women from ethnic minority backgrounds are at greater risk of adverse maternal outcomes. Antenatal care plays a crucial role in reducing risks of poor outcomes. The aim of this study was to identify, appraise, and synthesise the recent qualitative evidence on ethnic minority women’s experiences of accessing antenatal care in high-income European countries, and to develop a novel conceptual framework for access based on women’s perspectives.

**Methods:**

We conducted a comprehensive search of seven electronic databases in addition to manual searches to identify all qualitative studies published between January 2010 and May 2021. Identified articles were screened in two stages against the inclusion criteria with titles and abstracts screened first followed by full-text screening. Included studies were quality appraised using the Critical Appraisal Skills Programme checklist and extracted data were synthesised using a ‘best fit’ framework, based on an existing theoretical model of health care access.

**Results:**

A total of 30 studies were included in this review. Women’s experiences covered two overarching themes: ‘provision of antenatal care’ and ‘women's uptake of antenatal care’. The ‘provision of antenatal care’ theme included five sub-themes: promotion of antenatal care importance, making contact and getting to antenatal care, costs of antenatal care, interactions with antenatal care providers and models of antenatal care provision. The ‘women's uptake of antenatal care’ theme included seven sub-themes: delaying initiation of antenatal care, seeking antenatal care, help from others in accessing antenatal care, engaging with antenatal care, previous experiences of interacting with maternity services, ability to communicate, and immigration status. A novel conceptual model was developed from these themes.

**Conclusion:**

The findings demonstrated the multifaceted and cyclical nature of initial and ongoing access to antenatal care for ethnic minority women. Structural and organisational factors played a significant role in women’s ability to access antenatal care. Participants in majority of the included studies were women newly arrived in the host country, highlighting the need for research to be conducted across different generations of ethnic minority women taking into account the duration of stay in the host country where they accessed antenatal care.

**Protocol and registration:**

The review protocol was registered on PROSPERO (reference number CRD42021238115).

**Supplementary Information:**

The online version contains supplementary material available at 10.1186/s12913-023-09536-y.

## Background

Women from ethnic minority backgrounds in high-income countries are at greater risk of adverse maternal outcomes [1–3]. Maternal mortality in the United Kingdom (UK) between 2016–18 was highest among ethnic minority women and those of low socioeconomic status; among Black and Asian women, maternal mortality was respectively four and two times higher than among white women [3]. The reasons for this are multifaceted, with ethnic background intersecting with a range of contributory factors: socio-economic status, language, and culture, pre-existing physical and mental health conditions and differential access to and utilisation of health services [3, 4]. A high proportion of Black and Asian women did not initiate antenatal care by the recommended 10-week gestation or receive the full schedule of antenatal care [3]. Several studies from high-income countries have also shown delayed initiation of antenatal care among ethnic minority women [5–7].

The World Health Organization [8] urges that all women should have access to antenatal care which leads to a positive experience of pregnancy, and recommends that women receive eight antenatal appointments during pregnancy as a preventative measure against perinatal morbidity and mortality.

Previous systematic reviews have examined access to antenatal care for ethnic minority women in high-income countries. In an evidence synthesis exploring barriers to antenatal care for marginalised women in the UK, among which ethnic minority women were included, Downe et al. found that women’s access to antenatal care is highly contextualised, depending on their personal circumstances [9]. A subsequent review conducted by Hollowell et al. explored the initiation of, and interventions to, increase antenatal care, and reported multiple barriers to timely initiation of antenatal care for ethnic minority women in the UK, both at structural and individual levels, including additional barriers faced by asylum seekers [10]. The authors found that interventions (conducted mainly in the United States) to increase timely uptake of antenatal care addressed these barriers by focussing on outreach services and the provision of community or bicultural workers to advocate for women [10]. Focussing on maternity care more broadly rather than solely antenatal care, a review by Higginbottom et al. found that late initiation of antenatal care was a common theme among immigrant women’s narratives in the UK [11].

Given the continued poor maternity outcomes for ethnic minority women in high-income countries and the evidence suggesting delayed initiation and reduced uptake of antenatal care, there is an urgent need to update the evidence base, enabling organisational and clinical responses to be developed to shift these trends. This review was conducted to a) systematically synthesise qualitative evidence from studies conducted over the past decade (2010–2021) that have examined ethnic minority women’s experiences of accessing antenatal care in high-income European countries and b) to develop a novel conceptual framework to describe access to antenatal care for ethnic minority women in order to inform future interventions.

## Definitions of terms

For the purpose of this review, we used the term ‘ethnic minority’ to describe a group of people from a nationality or culture living in a geographical area where most people are from a different nationality or culture [12].

High-income European countries were identified based on The World Bank [13] classification (see Additional file 1 for a table of countries included).

Antenatal care is defined as “…the care provided by skilled health-care professionals to pregnant women and adolescent girls in order to ensure the best health conditions for both mother and baby during pregnancy.” (p.1) [8]. Routine packages of antenatal care, as a minimum, incorporate health education and promotion, the identification of risk and the management or prevention of illness and disease, during pregnancy [8].

Access to health care is complex and multifaceted and is described in more depth later in the paper. However, to summarise, we used the definition of access proposed by Santana et al.: “… getting the right health care in the right place at the right time.” ([14], p.7). We present the conceptualisation of access further in the paper when the “best fit” model of access to health care is discussed. The loci of focus in this systematic review is access to antenatal care, rather than antenatal care itself.

Qualitative research as described by Braun and Clarke as research that analyses “words which are not reducible to numbers” ([15], p.6) and which employs an inductive, non-positivist approach.

## Methods

The Preferred Reporting Items for Systematic Reviews and Meta Analyses (PRISMA 2021) guidelines [16] was used to conduct this systematic review. The review protocol was registered on PROSPERO (reference number CRD42021238115; https://www.crd.york.ac.uk/prospero/display_record.php?ID=CRD42021238115).

### Study inclusion and exclusion criteria

Qualitative and mixed-method studies were included if they explored and reported findings on ethnic minority women’s experiences of accessing antenatal care in high-income European countries and published in peer-reviewed journals between 2010 and 2021. Papers specifically exploring issues of access to antenatal care during the Covid-19 pandemic were excluded as this was not the focus of our review. A post-hoc exclusion criteria was developed during the abstract and article screening phase. Studies focussing only on specific components of antenatal care, such as antenatal screening tests or diagnosis of antenatal diseases were excluded from this review. The full inclusion and exclusion criteria are detailed in Table 1.

**Table 1:** Inclusion and exclusion criteria

**Inclusion criteria**	**Exclusion criteria**
1. Studies conducted in a high-income European country 2. Study participants included women from ethnic minority backgrounds 3. Aspects of accessing antenatal care explored 4. Qualitative or mixed-method data collected 5. Women’s perspectives reported in study findings 6. Studies published 2010–2021	**Pre-determined exclusion criteria:** 1. Studies conducted outside high-income European countries 2. Studies not published in English 3. Grey literature or studies published in non-peer reviewed journals 4. Studies reporting health professionals’, other professionals or father’s perspectives only 5. Studies focussed on access to antenatal care in relation to the Covid-19 pandemic 6. Studies published outside the search dates 7. Full text unavailable **Post-hoc exclusion criteria developed during abstract and article screening:** 1. Studies focussed solely on other aspects of maternity care (such as birth) 2. Studies focussed on aspects of antenatal screening, including screening diagnosis 3. Studies focussed on antenatal illness or disease (such as diabetes), or indicators of disease 4. Studies focussed on antenatal nutrition

### Study search strategy

The review question was framed using the PICOS framework: Population (ethnic minority women), phenomenon of Interest (access to antenatal care in high-income European countries), Comparator (none), Outcome (women’s experiences), and Study design (qualitative and mixed-methods studies). The corresponding search terms which we used can be found in Table 2.

**Table 2:** Key search terms

1	“Pregnancy” OR “pregnant” OR “pregnan” OR “prenatal” OR “antenatal” OR “perinatal “ OR “maternal” OR “mother” OR “women”	Population
2	“Ethnicity” OR “ethnic” OR “ethnic group” OR “ethnic minority” OR “ethnic minorities” OR “immigrant” OR “immigrants” OR “immigration” OR “migrant” OR “migrants” OR “migration” OR “emigrant” OR “emigration” OR “foreigners” OR “foreign nationals” OR “race “ OR “racial” OR “culture” OR “cultural” OR “custom” OR “Indigen” OR “indigenous” OR “ethnology” OR “minority group” OR “BAME”	
3	“Prenatal care” OR “antenatal care” OR “antepartum care” OR “during pregnancy care” OR “pregnancy care” OR “maternity care” OR “maternal care” OR “maternal healthcare” OR “maternal health perinatal care” OR “maternal health services” OR “health services” OR “women’s health services” OR “maternal health services” OR “health-services indigenous” OR “obstetric-care-trends” OR “women's health-trends” OR “transcultural care” OR “prenatal care-trends” OR “booking”	Phenomena of Interest
4	“Acceptability” OR “accept” OR “access” OR “attend” OR “utilization” OR “uptake” OR “use” OR “obtain” OR “visit” OR “appointment” OR “adherence”	
5	“Experiences” OR “perceptions” OR “attitudes” OR “views” OR “feelings” OR “perspectives” OR “expect” OR “anticipate” OR “encounter” OR “belief” OR “exploration”	Outcomes
6	1 AND 2 AND 3 AND 4 AND 5	

Two authors (ES and PT) conducted the search. The following databases were searched between 18^th^ January and 12^th^ February 2021: PubMed (National Library of Medicine), PsycINFO (Ovid), SCOPUS (Elsevier), CINAHL Complete (EBSCOhost), Medline (EBSCOhost), Global Health (EBSCOhost) and Cochrane (Wiley). A manual search of reference lists from primary studies was conducted to include a diverse and rich sample of relevant studies across a range of settings and groups. Titles and abstracts were double-screened for eligibility (ES and PT). Where there were uncertainties, the full text was obtained. Any disagreements between those screening were discussed with the third author (SP).

### Data extraction and quality assessment

Study characteristics were extracted from the included studies (ES and PT) by study aim, setting, participants, data collection methods, method of analysis and main findings. Quality assessment of studies was conducted using the Critical Appraisal Skills Programme [17] tool, by two reviewers (ES and PT). No studies were excluded based on their quality, as it is recognised that even poor quality qualitative studies can yield useful findings [18].

### Evidence synthesis methods

**Fig. 1:**
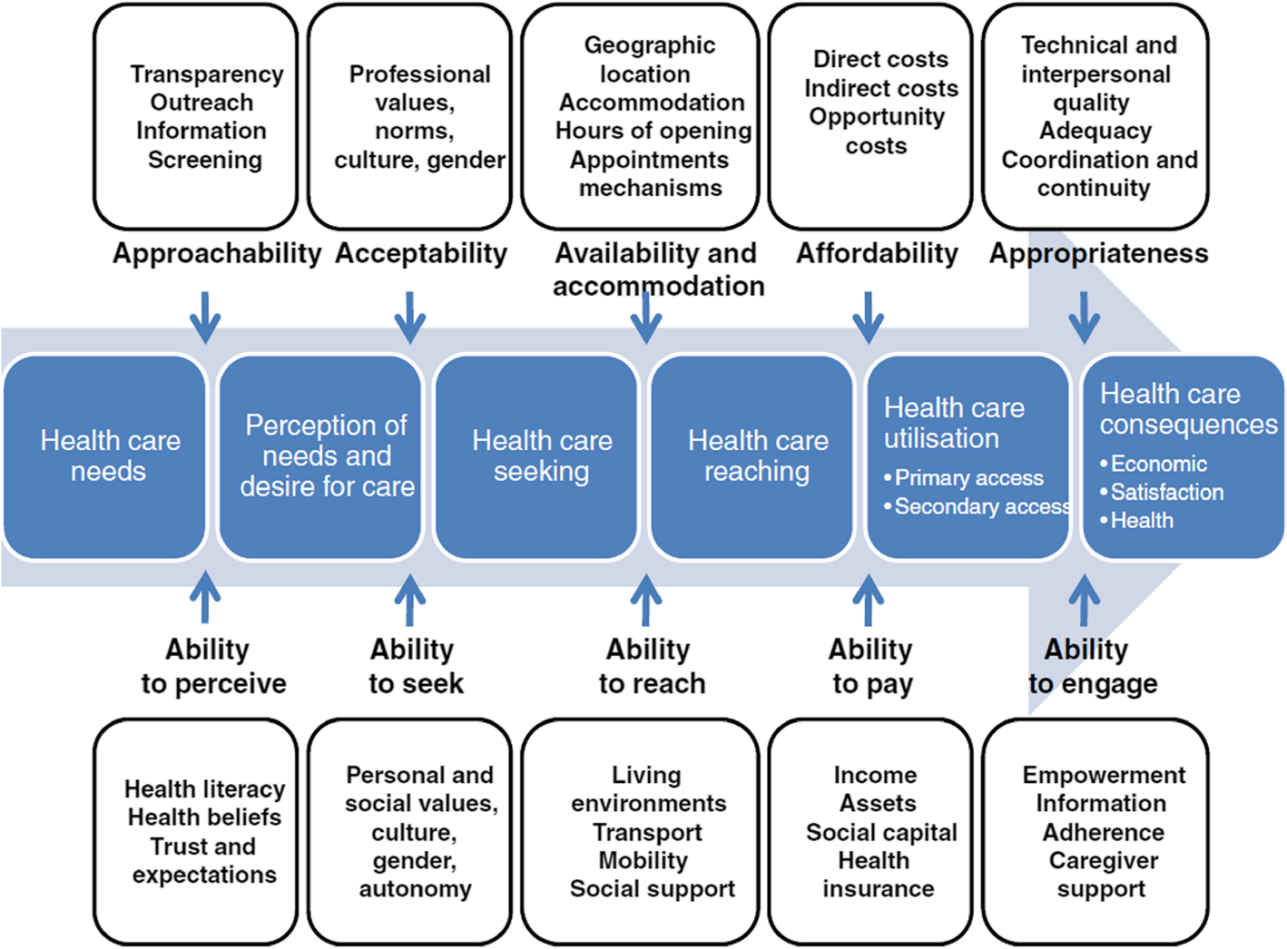
A conceptual framework for access to health care. Reproduced with permission from Levesque et al. [21]

The “best fit” framework synthesis approach was chosen to guide this review, following the stages set out by Carroll et al. [19]. This approach to evidence synthesis combines existing theoretical frameworks with current evidence to develop a novel conceptual framework to explain behaviours or decision-making among populations [20]. Knowing that there was an existing corpus of literature and conceptual frameworks regarding access to care, but not specifically related to ethnic minority women’s access to antenatal care, we chose to use “best fit” framework synthesis because it is a method of qualitative evidence synthesis which offers the opportunity “to test, reinforce and build on an existing published model” ([20], p.1). The initial step in this approach was the development of a theoretical framework which formed the a priori “best fit” framework against which data could be deductively coded. We identified Levesque’s model of access to health care [21] which was developed through a process of systematic identification and synthesis of access to health care models and was therefore suitable for using as a “best fit” model (Fig. 1). Levesque’s model of access to health care is divided into supply-side factors (the accessibility of services, including approachability, acceptability, availability and accommodation, affordability and appropriateness) and demand-side factors (the ability of individuals, populations or communities to “interact with the dimensions of accessibility to generate access” ([21], p.5), including ability to perceive, ability to seek, ability to reach, ability to pay and ability to engage).

The second step in the “best fit” framework synthesis approach was to code the data. Findings from included studies were exported into NVivo v.12 [22]. All qualitative study findings were coded deductively against a priori themes, drawn from the ‘best fit’ framework [20]. Thematic analysis [23] was used to code data that could not be assigned to the a priori themes. In the final step, all a priori*,* and new themes and sub-themes were amalgamated to reflect the dataset and we returned to the evidence to look for relationships between these themes in order to develop the new conceptual model. Coding was conducted by the first author (ES) with ongoing discussion with the second and third authors (SP and PT).

## Results

### Included studies

**Fig. 2:**
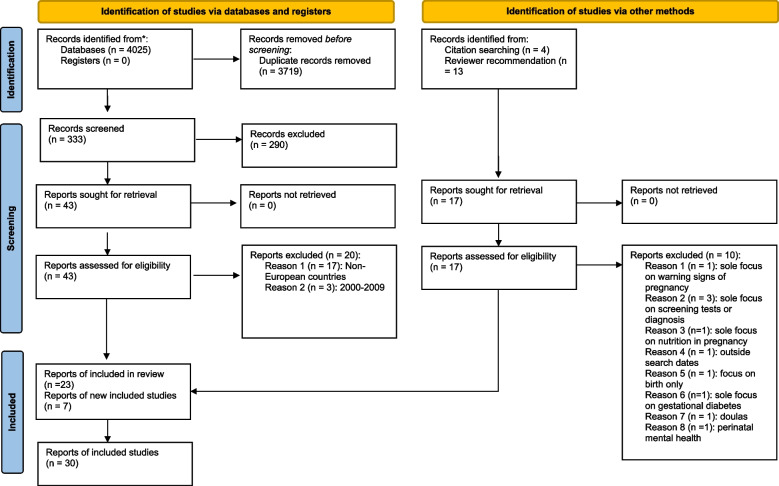
PRISMA flow chart of the study selection process

A total of 4052 studies were identified though the electronic search, an additional four studies from the manual search and a further three were added after the review process. After removing duplicates, 337 records were screened for eligibility and 30 studies were included (Fig. 2).

Studies were conducted in 11 different countries. The majority of studies were conducted in the UK (*n* = 12) [24–35]; other studies were conducted in Portugal (*n* = 5) [36–40], Sweden (*n* = 3) [41–43], Switzerland (*n* = 2) [44, 45], Finland (*n* = 2) [46, 47], Belgium (*n* = 1) [48], Germany (*n* = 1) [49], Denmark (*n* = 1) [50], Netherlands (*n* = 1) [51], Norway (*n* = 1) [52] and Spain (*n* = 1) [53]. Study participants represented a diversity of ethnic backgrounds. In 12 studies, participants were identified as recent arrivals (arrived in the past 5 years) in their host country. Only two studies explicitly stated that participants included women of ethnic minority backgrounds who were born in the country of study [24, 26]. Study characteristics are summarised in Table 3.

**Table 3:** Summary of study characteristics

**Authors (year)**	**Aim**	**Study setting**	**Data collection**	**Participants**		**Data Analysis**	**Main themes**
				**Recruitment**	**Country of origin**		
Puthussery et al. (2010) [24]	To explore the maternity care experiences and expectations of United Kingdom (UK)-born ethnic minority women	UK	Semi-structured interviews (SSI)	Recruitment method not stated (*n* = 34)	UK-born Indian, Pakistan, Bangladeshi, Black African, Black Caribbean	Grounded theory—open coding using NVivo	Equitable care; Sensitive care; Continuity of care; Access to information and communication; Care environment
Binder et al. (2012a) [28]	To identify delay-causing influences on the pathway to optimal facility treatment	UK	SSI and focus group discussions (FGD)	Snowball sampling of immigrant sub-Saharan African women (*n* = 54); purposive sampling of NHS maternal providers (*n* = 62)	Sub-Saharan Africa	Intuitions and triangulation of findings using constructivist hermeneutic approach	Delays at the facility level due to lack of mutual trust between women and maternal care; Unmet expectations hindering providers from delivering optimal maternal healthcare
Binder et al. (2012b) [25]	Communication and conceptions of obstetric care that immigrant women experience in a multi-ethnic obstetric care setting	UK	In-depth interviews (IDI) and FGD	Snowball and purposive sampling of Somali, Ghanaian and white British service users (*n* = 60) and maternity care providers (*n* = 62)	Somalia, Ghana, White British	Intuitive categories generated from findings	Language; Interpreter as a tool for communication; Cultural influences on communication
Wikberg et al. (2012) [47]	To describe and interpret the perceptions and experiences of caring of immigrant new mothers in maternity care	Finland	Interviews, unstructured observations and ‘other documents’	Convenience sampling (*n* = 17)	Various	Not clear	Tension between the expectations of the mothers and their Finnish maternity care experience of caring; Caring Was related to the changing culture; Finnish maternity care traditions were imposed on the immigrant new mothers influencing caring; Female nurse was seen as a professional friend; Conflicts encountered were resolved, which promoted caring
Alshawish et al. (2013) [29]	To investigate access to and use of maternal and child health services	UK	SSI	Convenience and snowball sampling (*n* = 22)	Palestine	Framework analysis	Cultural variations; Knowledge of the NHS and the UK health-care system; Health-care services and their utilisation, focusing on maternal and child health-care services; Communication; Information provision and needs
Barona-Vilar et al. (2013) [53]	To explore the experiences and perceptions of parenthood and maternal health care among Latin American women living in Spain	Spain	FGD	Convenience sampling; Latin American women (*n* = 26); Spanish midwives (*n* = 24)	Bolivian and Ecuadorian mothers; Spanish midwives	Content analysis	Cultural significance of parenthood; Planning for motherhood; Maternity and health care; Support networks during maternity
Almeida and Caldas (2013) [40]	To assess possible differences regarding women's perceptions regarding the quality and appropriateness of maternal health care received	Portugal	SSI	Purposive sampling of migrant women (*n* = 14)	Brazil, Portugal	Content analysis	Health status; Perceptions of access and quality of care, in comparison with those in the country of origin; Barriers to and facilitators of the use of healthcare services; Perceived gaps in the health system and suggestions for improvement; Mother and child healthcare; Strategies for managing difficulties; Quality and consequences of the care provided by health professionals; Contraception—information, decision and use
Almeida et al. (2014a) [37]	To identify and understand patterns of satisfaction and demand of maternal and child healthcare, assessing women’s perceptions about its quality	Portugal	SSI	Sampling method not stated (*n* = 31)	Portuguese-speaking African countries, Eastern European Countries, Brazil, Portugal	Content analysis	Maternal and child healthcare; Strategies for managing difficulties; Quality and consequences of care by health professionals
Almeida et al. (2014b) [38]	To provide qualitative information on the access, use and perceived quality of care during pregnancy and early motherhood	Portugal	SSI	Sampling method not stated (*n* = 31)	Portuguese-speaking African countries, Eastern European Countries, Brazil, Portugal	Content analysis	Health status; Access and quality of care, in comparison with the country of origin; Barriers and facilitators for the use of healthcare services; Perceived problems in the health system and suggestions for improvement
Degni et al. (2014) [46]	To explore immigrant Somali women's experiences of reproductive and maternity health care services (RMHCS) and perceptions about service providers	Finland	FGD	Purposive sampling (*n* = 70)	Somalia	Not stated	Women’s experiences of RMHCS; Women’s perceptions about cultural and communication competencies of the health care professionals providing these services
Coutinho et al. (2014) [36]	To identify the unmet expectations of Portuguese and immigrant women, for the National Health System, during pregnancy, childbirth and postpartum	Portugal	Semi-structured interviews (SSI)	Recruitment method not stated (*n* = 82)	22 Portuguese and 60 immigrants, belonging two to Clusters of Health Centres (ACES) in the districts of Viseu and Aveiro	The Technique of Categorical Content Analysis- QSR NVivo version 10	Incentives to maternity; the accessibility; human resources; physical and environmental conditions; organization of the health system
Robertson (2015) [42]	To analyse women's reflections on how their migration and resettlement experiences influenced health and healthcare needs during childbearing	Sweden	FGD and narrative interviews	Purposive sampling (*n* = 25)	Bosnia, Chile, China, El Salvador, Ethiopia, Eritrea, Iran, Iraq, Kosovo, Lebanon, Morocco, Slovenia, Spain, Syria, Turkey, Uzbekistan, and former Yugoslavia	Content analysis	Migrating can both bring relief and cause grief; Resettling takes a lot of effort; Vulnerability in childbearing encounters; Tensions in the body
Phillimore (2015) [31]	To understand migrants women’s experiences of seeking maternity care	UK	Semi-structured questionnaire, narrative interviews and SSI	Snowball sampling for questionnaire (*n* = 44); convenience sampling for interviews (*n* = 13)	28 different countries (countries not stated)	Systematic thematic analysis	Language and communication; Cultural health capital and discrimination; Power and control; Structural inequalities; Social networks and communications
Moxey and Jones (2016) [32]	To explore how Somali women exposed to female genital mutilation experience and perceive antenatal and intrapartum care in England	UK	SSI	Snowball & convenience sampling (*n* = 10)	Somalia	Thematic analysis	Experiences of FGM during life, pregnancy and labour; Experience of care from midwives; Adaptation to English life
Lephard and Haith-Cooper (2016) [33]	To explore the maternity care experiences of local, pregnant, asylum-seeking women	UK	SSI	Purposive and snowball sampling (*n* = 6)	sub-Saharan Africa, Eastern Europe	Thematic analysis	Pre-booking challenges; Inappropriate accommodation; Being pregnant and dispersed; Being alone and pregnant; Not being asked or listened to
Phillimore (2016) [30]	To explore the reasons new migrant women book late and do not attend antenatal follow-up appointments	UK	Questionnaires and IDI	Snowball sampling for questionnaires (*n*- = 82); sampling methods for interviews with migrant women (*n* = 13); convenience and snowball sampling for interviews with professionals (*n* = 18)	Not stated	Systematic thematic approach	Initial booking: Lack of flexibility in the system; Basic information about procedures and routines was not readily available to migrant women and rarely translated into new migrant languages; Lack of knowledge about pregnancy monitoring processes; Professionals struggled to get hold of interpreters to enable a proper assessment Follow-up appointments: Non-attendance at follow-up appointments after booking; Lack of flexibility and choice; Low income; Felt attendance was pointless
Topa et al. (2017) [39]	To explore the engagement, perceptions and experiences of Ukrainian immigrant women, on maternal and child healthcare services	Portugal	SSI	Purposive sampling (*n* = 10)	Ukraine	Thematic analysis then critical discourse analysis	Diaspora; Maternal care: facilities and constraints
Barkensjö et al. (2018) [41]	To provide a composite description of undocumented women’s experiences of clinical encounters throughout pregnancy and childbirth	Sweden	Unstructured interviews	Sampling method not stated (*n* = 13)	Macedonia, Romania, Bosnia, Albania, Somalia, Afghanistan, Serbia, Chechnya, Morocco, and Kosovo	Content analysis	Experiencing acute distress when suffering subpar and neglectful clinical encounters; Feeling acknowledged and empowered through experiencing positive clinical encounters
Origlia Ikhilor et al. (2019) [44]	To describe the communication barriers of allophone migrant women in maternity care	Switzerland	FG and interviews	Convenience sampling (*n* = 34)	Eritrea, Albania, Kosovo; Swiss healthcare professionals; intercultural interpreters (ethnicity not stated)	Thematic analysis	Circumstances and healthcare system; Relations and interactions; Adaptation of health services
Sami et al. (2019) [45]	To explore experiences with, and barriers to, maternal health services	Switzerland	FGD	Convenience and snowball sampling of migrant women (*n* = 33)	Portugal Peru, USA, Germany Dominican Republic, Peru, Bolivia Brazil Eritrea Bangladesh	Coded and topics and sub-topics identified	Good maternity services; Barriers on the patient side; Barriers on the maternity service level
Peters et al. (2019) [51]	To gain an understanding of the obstetric healthcare system as experienced by low-educated native Dutch and non-western ethnic minority women	Netherlands	FGD	Purposive and snowball sampling (*n* = 106)	Turkey, Morocco, Surinam, Antilles, Cape Verde, native Dutch, various other ethnic backgrounds	Direct content analysis and variable-oriented analysis	Autonomy; Communication; Confidentiality; Dignity; Prompt attention; Quality of basic amenities; Choice; Access to family and community support
Chinouya and Madziva (2019) [34]	To explore why Black African women access the initial antenatal after 13 weeks of pregnancy in a London borough	UK	SSI	Purposive and snowball sampling (*n* = 23)	Ghana, Kenya, Uganda, Nigeria, Somali, Zimbabwe	Framework analysis	Unresolved immigration status; Importance of culture; Lack of awareness; Cultural expressions of pregnancy
Ahrne et al. (2019) [43]	To explore Somali-born parents’ experiences of antenatal care in Sweden and antenatal care midwives’ experiences of caring for Somali-born parents, and their ideas about group antenatal care for these women	Sweden	FGD	Purposive sampling Somali-born parents (*n* = 29) and midwives (*n* = 7)	15 born in Somalia and 1 born in Sweden	Attride-Stirling’s tool “Thematic networks”	Challenges in the midwife-parent encounter: Tailoring care to individual needs; Dealing with stereotypes; Addressing varied levels of health literacy; Overcoming communication barriers and partner involvement Health system challenges: Accessibility of carer; Clear but flexible routines; Limited resources and supportive structures for parent education
Hassan et al. (2019) [26]	To investigate Muslim women’s perceived needs and the factors that influence their health seeking decisions when engaging with maternity services located in North-West of England	UK	A qualitative longitudinal in-depth SSI	Purposeful sampling (*n* = 21)	UK born (4) and born outside the UK (3), housewives (4), employed (3), married (7), ethnicity; Yemeni (3), White British (2), Somali and British Indian, and with secondary school as minimum level of education (7)	Thematic analysis	A spiritual perspective; Expression of religious requirements; Perceptions of healthcare professionals
Henry et al. (2020) [49]	To investigate how premigration experiences, conceptions, health literacy and language skills influence access to and experiences of maternity care	Germany	Interviews	Sampling method not stated but appears to be convenience (*n* = 12)	Iraq, Syria, Palestine	Content analysis	Perceptions of health care needs; Health care seeking; Health care consequences; Health-care experiences during pregnancy and childbirth; Compensation mechanisms
Filby et al. (2020) [27]	To evaluate the specialist migrant maternity service provided by a London hospital based upon users' experience and satisfaction	UK	SSI	Purposive sampling (*n* = 10)	Vietnam, China, Albania, Nigeria, Afghanistan, Yeman	Thematic analysis	Accessibility of the midwife and referrals; Provision of essentials and transport; Respect and kindness; Lack of nutritious diet; Lack of facilities for hygienic formula preparation and storing breast milk; Lack of signposting to services; Lack of social support building
Ceulemans et al. (2020) [48]	To explore the experiences of Arabic-speaking pregnant migrant women living in Belgium regarding communication and perceptions towards health professionals and use of healthcare products during pregnancy	Belgium	SSI and questionnaire	Purposive and snowball sampling (*n* = 17)	Arabic-speaking women: Syria, Palestine and others (not stated)	Qualitative Analysis Guide of Leuven (QUAGOL)	Communication with healthcare professionals: Language barriers; Current communication strategies with HCPs; Future strategies to improve communication with HCPs Perceptions about healthcare professionals and healthcare products: Perceptions about healthcare professionals; Perceptions about the use of healthcare products during pregnancy
Funge et al. (2020) [50]	To explore undocumented immigrant women’s experiences of, as well as their access to, maternity care services during pregnancy in Denmark	Denmark	SSI	Purposive sampling (*n* = 21)	Philippines, Sudan, Morocco, Pakistan, Kenya, Tanzania, Uganda, Bosnia	Malterud’s guidelines for systematic text condensation	Access to public maternity care services; Use of private and non-governmental maternity care services; Perception of entitlements to care; Being dependent
Utne et al. (2020) [52]	To explore Somali women's experiences of antenatal care in Norway	Norway	SSI	Snowball sampling (*n* = 8)	Somalia	Systematic text condensation	When care was provided in a way that gained their trust, they made better use of the available health services; The importance of continuity of care and of sharing commonalities with the caregiver; A need for accessible information, specifically tailored to the needs of Somali women; How culturally insensitive caregivers had a negative impact on the quality of care
Nellums et al. (2021) [35]	To investigate experiences of undocumented migrant women who have been pregnant in England, and factors affecting access to care and health outcomes	UK	SSI	Purposive and snowball sampling (*n* = 20)	Various (not stated)	Thematic analysis	Restricted agency; Intersecting stressors; Ongoing cycle of precarity

### Quality of included studies

The quality appraisal results of the 30 included studies are summarised in Table 4. Research aims were clearly addressed in all studies. Over half (*n* = 16) of the studies adequately discussed the choice of research design. Sufficient information about recruitment (*n* = 11) [26, 27, 29, 34–36, 41–43, 49, 50] and methods of data collection (*n* = 13) [24, 29, 32, 35–37, 40–43, 46, 49, 52] were less commonly reported. Issues of reflexivity and how the researcher role impacted the study were only addressed in 2 studies [34, 35]. Ethical considerations were discussed in one third of the studies (*n* = 10) [24, 26, 27, 33, 36, 41–43, 49, 50]. Although data analysis was presented in most studies, few provided adequate details to determine its rigor (*n* = 6) [29, 32, 35, 43, 51, 52]. A small number of studies (*n* = 10) did not discuss findings in relation to current policy and practice and how they could be implemented [24, 25, 29, 30, 37–40, 46, 47].

**Table 4:** Methodological quality assessment using the CASP checklist

**First author (year)**	**Research aims?**	**Methodology?**	**Research design?**	**Recruitment?**	**Data collection?**	**Reflexivity?**	**Ethical issues?**	**Data analysis?**	**Statement of findings?**	**How valuable?**
Puthussery et al. (2010) [24]	√	√	√	?	√	×	√	?	×	√
Binder et al. (2012a) [28]	√	√	?	?	?	×	×	?	√	√
Binder et al. (2012b) [25]	√	√	√	?	?	×	×	×	×	√
Wikberg et al. (2012) [47]	×	√	?	?	?	×	?	?	×	?
Alshawish et al. (2013) [29]	√	√	√	√	√	?	?	√	×	√
Barona-Vilar et al. (2013) [53]	√	√	√	?	?	×	×	?	√	?
Almeida and Caldas (2013) [40]	√	√	√	?	√	×	×	?	×	?
Almeida et al. (2014a) [37]	√	?	?	?	√	×	×	?	×	?
Almeida et al. (2014b) [38]	√	√	?	?	?	×	×	?	×	?
Degni et al. (2014) [46]	√	√	?	?	√	×	×	?	×	?
Coutinhoa et al. (2014) [36]	√	√	√	√	√	×	√	?	√	√
Robertson (2015) [42]	√	√	√	√	√	×	√	?	√	√
Phillimore (2015) [31]	√	√	?	?	?	×	×	?	√	√
Moxey and Jones (2016) [32]	√	√	?	?	√	×	?	√	√	√
Lephard and Haith-Cooper (2016) [33]	√	√	√	?	?	×	√	?	√	√
Phillimore (2016) [30]	√	√	√	?	?	×	×	?	×	√
Topa et al. (2017) [39]	√	√	?	?	?	×	?	?	×	√
Barkensjö et al. (2018) [41]	√	√	√	√	√	×	√	?	√	√
Origlia Ikhilor et al. (2019) [44]	√	√	√	?	?	?	?	?	√	?
Sami et al. (2019) [45]	√	√	?	?	?	?	?	?	√	√
Peters et al. (2019) [51]	√	√	?	?	?	×	?	√	√	√
Chinouya and Madziva (2019) [34]	√	√	√	√	?	√	×	?	?	√
Ahrne et al. (2019) [43]	√	√	√	√	√	?	√	√	√	√
Hassan et al. (2019) [26]	√	√	√	√	?	?	√	?	?	√
Henry et al. (2020) [49]	√	√	?	√	√	×	√	?	√	?
Filby et al. (2020) [27]	√	√	?	√	?	?	√	?	√	√
Ceulemans et al. (2020) [48]	√	√	?	?	?	×	?	?	√	√
Funge et al. (2020) [50]	√	√	√	√	?	×	√	?	√	√
Utne et al. (2020) [52]	√	√	√	?	√	×	?	√	√	√
Nellums et al. (2021) [35]	√	√	?	√	√	√	?	√	√	√

^*^Yes: √; No: × ; Can’t tell: ?

### Evidence synthesis

In keeping with the ‘best fit’ framework from Levesque et al. [21], themes were broadly grouped in two: theme 1, *Provision of Antenatal Care* (“supply side”) and theme 2, *Women's Uptake of Antenatal Care* (“demand side”). Theme 1 comprised of five sub-themes: 1. promotion of antenatal care importance, 2. making contact and getting to antenatal care, 3. costs of antenatal care, 4. interactions with antenatal care providers and 5. models of antenatal care provision. Theme 2 comprised of seven sub-themes: 1. delaying initiation of antenatal care, 2. seeking antenatal care, 3. help from others in accessing antenatal care and 4. engaging with antenatal care. The remaining three sub-themes cut across all theme 2 sub-themes insofar as they impacted access to antenatal care as they were reflected in all the aforementioned sub-themes: 5. previous experiences of interacting with maternity services, 6. ability to communicate and 7. immigration status. The themes are discussed in detail below.

1. Provision of antenatal care
  1.1 Promotion of antenatal care importance
  One study [34] reported perspectives about lack of the promotion of antenatal care in public places. The participants noted promotion about the importance of early initiation of antenatal care as a key element towards making antenatal care more accessible:
    “*I think the benefits of booking early need to be advertized, I have never seen that anywhere like bus stops, or having leaflets or posters. Not everyone has access to technology*.” ([34], p.128)
  1.2 Making contact and getting to antenatal care
  Participants in eight of the included studies [29–31, 42, 43, 45, 50, 53] identified that the mechanisms for making antenatal appointments could be problematic. Women often found the logistics of appointment-booking difficult and time consuming [29, 41, 42], appointment schedules inflexible [50, 53], and experienced delays in obtaining an appointment [29, 36].
    “*Yes, we have to wait a long time… for care and there [in Ukraine] it is not so. I was not expecting it to be so*” ([36], p.141)
    In six studies [25, 27, 30, 31, 43, 50], geographical location of appointments was significant for women in accessing antenatal care, linked to which was the expense and logistics of transportation to appointments located further away [30, 31, 50]. One participant here describes the impact that geographical location could have on her ongoing access to antenatal care:
    “*I want to come as the midwife has told me, but sometimes it is just not possible. It is too far and costs too much. I can’t do anything about it. I have to come some other time then*” ([50], p.6)
  1.3 Costs of antenatal care
  Issues relating to costs of antenatal care were discussed in six studies [29, 35, 38, 50, 52, 53]. Two of these reported women’s expressions of gratitude for maternity care provided free at the point of use [29, 52]. However, the provision of free antenatal care was more frequently framed in the context of insufficient information about entitlement to state-provided antenatal care among migrant women [50, 53] and their difficulties navigating access to it [35, 38, 50, 53]. In countries with policies of restrictive access to health for some migrant groups, some women spoke of their shock and unpreparedness for receiving bills for their care, the impact of which could create a barrier to accessing antenatal care [35, 50], as exemplified here:
    “*Just last month I’ve just received a bill…£5654…we don’t have any income, nothing…No one told me that, ‘We are charging you for that*’.’” ([35], p.4).
  1.4 Interactions with antenatal care providers
  In six studies [27, 37, 38, 41, 44, 52], the role which antenatal care providers played in initial and ongoing access was apparent. There were mixed views regarding the extent to which a care provider from a women’s own ethnic background was important, some stating that it is of no consequence [24, 28] and others emphasising the language benefits and cultural understandings that a care provider from the same ethnic background could bring [28, 29].
  Stigmatising and discriminatory interactions with care providers were frequently experienced by women [33, 35, 42, 43, 52], as illustrated by a Somali woman here:
    “*When she (the midwife) was talking to me, I felt like there wasn’t a great deal of respect. I felt as though she was thinking “you stupid Somali mothers*”.” ([52], p.4).
  Negative experiences could impact further access to antenatal care, as will be discussed later.
  1.5 Models of antenatal care provision.
  In this review, models of antenatal and maternity care provision (including models such as physician-led care, midwifery-led care, care shared between general practitioners and midwives, and midwifery continuity of carer) intersected with access to antenatal care at every stage, as seen in seven of the included studies [24, 27, 30, 42, 45, 47, 52]. Where provided, specialist maternity care for migrant women facilitated a greater ease for women in making contact with care providers [27, 45]. For example, in one study, asylum seeking women living in a UK emergency accommodation facility received on-site specialist midwifery care which enabled these women to access midwives easily [27]. The continuity of carer provided by this service was highly valued by women (ibid.), as it was by other women who received continuity of carer in another study [51]. Participants who did not have continuity of carer often expressed a desire for a known caregiver [24, 30, 42, 45]:
    “…*because it wasn’t like you had one midwife who knew you, who knew your problems, who knew you from the beginning, it wasn’t like that. And that would have helped*.” ([24], p.9)
  Continuity was valued by study participants because it fostered trust, a sense of safety and a supportive relationship.

2. Women’s uptake of antenatal care
  2.1 Delaying initiation of antenatal care
  In four studies, reasons for delaying initiation of antenatal care were identified [29, 34, 44, 53]. For some women, this was due to their cultural beliefs about early pregnancy [29, 34]. In particular, Black African women stated their belief that talking about their pregnancy in the early stages could be dangerous, as described here:
    “*You cannot speak about pregnancy before a certain period, 13 weeks they say, some evil people can say a bad word [cursing] and your pregnancy may abort [miscarriage] so you have to keep quiet*.” ([34], p.129).
  For other women there was a perception that antenatal care is not important [50, 53]. One parous woman said,
    “… *with my son I had no problems, well I said “With my daughter I can have her without anyone giving me medical care during my pregnancy*.”” ([53], p.335).
  Nonetheless, study participants held beliefs and perceptions which may have resulted in both delaying access to antenatal care as well as talking about the desire to take care of themselves and their baby during pregnancy [34, 50]. One study identified Muslim faith as a factor which may positively encourage women to seek health care,
    “… *as it is a religious duty to look after one’s self and this does not contradict reliance on Allah or the acceptances of one’s fate.*” ([26], p.5).
  2. 2 Seeking antenatal care
  Women reported difficulties in understanding how to navigate access to antenatal care in eight of the included studies [30, 32, 33, 35, 37, 38, 41, 44]. This was marked by difficulties in locating the information required, understanding the referral pathways to antenatal care and knowledge about how the overall health system works [32, 37, 38, 41, 47]. One Somali woman described this saying,
    “… *as a community we get very worried about things because we don’t really know much about the antenatal care services in England, we don’t know how things work here… it is something that is mentioned a lot within the community*.” ([32], p.6)
  Additionally, negotiating health system bureaucracy was found to be challenging [37, 45, 50, 53], for example:
    “*At the Health Centre I was told that I could not do my pregnancy follow-up there, because I changed my address. I was already three months pregnant! …in the new Health Centre, I wasn’t accepted*…” ([37], p.337).
  Conversely, ethnic minority women who were already familiar with health systems had no difficulties in this aspect of access to antenatal care [24, 32].
  Living environments were seen in this review to adversely impact access to antenatal care for some categories of migrant women, particularly with respect to housing provision, homelessness and government policies of dispersal [30, 31, 33, 35]. One undocumented migrant woman in the UK described how her homelessness affected her access to antenatal care:
    “… *I was sleeping rough outside…When I went [to the hospital] five months pregnant, they were asking me, ‘Have you got any scan?’ I said, ‘No.’…I couldn’t manage anything*.” ([35], p.4)
  Employment flexibility also impacted access to antenatal care. Women reported fear of losing employment when requesting time off to attend appointments, particularly when working illegally or on short-term contracts [30, 31, 39, 50, 53]. Ahrne et al. [43] found that appointment flexibility was deemed to be desirable for women.
  2.3 Help from others in accessing antenatal care
  In four studies, access to antenatal care was assisted by others [30, 41, 44, 50]. A number of women received assistance in accessing antenatal care from their partner [50], friends and family [32, 35, 39, 46], or non-governmental organisations [41, 50].
  2.4  Engaging with antenatal care
  While engaging with antenatal care, women were keen to understand how to stay healthy in pregnancy and which foods to avoid, as well as understanding medical tests and procedures [34, 50, 52]. However, six studies found that the information needs of participants were not met [24, 31, 45, 47, 51, 52], resulting for some women in a withdrawal from engagement with antenatal care, seeking information instead from informal sources [49, 50, 52]:
    “*I asked questions like if it was OK to eat fish and stuff. The midwife didn’t answer me, but told me to go home and read the brochures and take better care of myself. (…) I therefore pulled away*…” ([52], p.3).
    Support from antenatal care providers, including care providers’ *“…kindness, curiosity, commitment, and a warm and welcoming encounter…” ([*43*], p.109)* was highly valued by women and resulted in women being able to build a trusting relationship [41–43]. In two studies [42, 52], caregivers were a source of support for women with limited social networks. Participants commented that they were more likely to attend antenatal care if they had a good relationship with their care provider [41].
  The following three sub-themes cut-across all seven of the Theme 2 sub-themes.
  2.5 Previous experiences of interacting with maternity services
  Past experiences of interacting with maternity services during the current or previous pregnancy, or interacting with health services more broadly, were seen in five studies [25, 30, 42, 44, 52] as impacting access to antenatal care at all stages, including initiation and continuation of antenatal appointments. Women in a number of studies expressed overall satisfaction with their care [27, 38, 45, 46], but poor experiences in the form of stigmatisation, communication difficulties, or being denied care, were also evident, which resulted in a breakdown in trust and avoidance of attending further antenatal care [25, 30, 42, 44, 52]. This was exemplified in the account from a Somali woman, who relays what she was told by a health professional here:
    “ ‘*Why do you have another child? It would be better if you finished breastfeeding first, so that the children are not so close.’ I can’t put up with such information. I refuse to listen to it. That’s the reason I don’t want to go there*… “([43], p.110).
  2.6  Ability to communicate
  Language barriers permeated all aspects of access to antenatal care, as identified in 14 of the included studies, from navigating health systems to engaging with antenatal care providers [25, 27, 29, 30, 33, 38, 40, 41, 45, 46, 48, 49, 51, 53]. Language barriers hindered women’s communication with antenatal care providers [25, 29, 45, 48, 52] and study participants spoke repeatedly of the lack of suitable interpreters [25, 27, 29, 30, 33, 40, 41, 45, 46, 50, 51, 53]. Additionally, participants spoke of a lack of written information translated into their language [30, 45, 48, 51]. One study reported a participant’s view of delaying antenatal care until a doctor was available who spoke her language:
    “*Sometimes I wait 4–5 days to have an appointment with the GP, because I want an Arabic doctor to see me. My English language is poor and I feel more comfortable when I deal with an Arabic doctor. He can understand me*.” ([29], p.575)
  In another study, it was found that participants ceased attending appointments when language needs were not met [30].
  2.7 Immigration status
  A further sub-theme which was found to cut across all aspects of antenatal care is that of the impact of precarious immigration status, described in eight studies [30, 31, 33–35, 41, 44, 50]. Alongside parents expressing the need for antenatal care not being linked to their migrant status, rather to their individual background, a frequent finding among this group of participants was fear of being reported to immigration authorities and deported, or having children removed from them if antenatal care was accessed [30, 34, 50], as described here:
    “… *it used to be easy to just register at the GP’s but now they say, you want to register, bring your passport. And if you don’t have paper, you are scared that they will call those people [immigration officials] on you*… “ ([34], p.126).
  This could result in delaying seeking antenatal care, as the complexities of quotidian life took priority [30, 33].

### Conceptual model

**Fig. 3:**
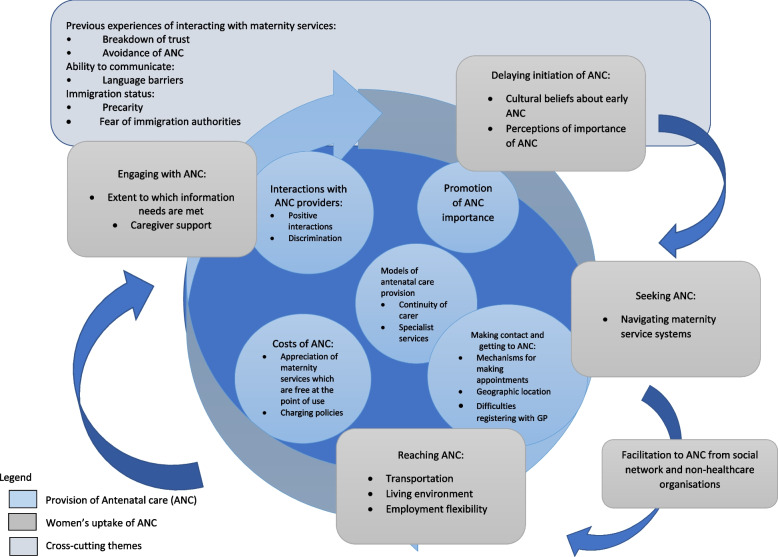
Conceptual model for access to antenatal care for ethnic minority women in high-income European countries

The novel conceptual model that we developed (Fig. 3) from updating the ‘best fit’ model with the evidence synthesis findings, illustrated the overlapping and interrelated nature of access to antenatal care for ethnic minority women. It was developed iteratively, going back and forth between the ‘best fit’ model and the synthesised data to ensure it adequately reflected the evidence, and went some way to bridging the gap between academic knowledge and real-world application [20]. The centre of the model comprised of five dimensions of access to care from the “supply side”, including the factors influencing the manner in which antenatal care was provided. The “demand side” dimensions of access to antenatal care, which included the factors influencing women’s ability to take up antenatal care, are shown on the outside of the model, yet overlapping with the “supply side” (provision of antenatal care) dimensions. Unlike it was depicted in the original “best-fit” model that we used, which represented access to health care in a linear format, we found that access to antenatal care could be represented better in a cyclical pathway, with previous experiences of accessing antenatal care (or indeed health care more broadly) impacting future access to antenatal care, whether in the existing, or a new pregnancy (as illustrated by the thin blue arrows on the diagram). This in turn would add to an understanding that access to antenatal care was not a one-off event, but rather it was an event with a beginning and ongoing occurrence once begun, both of which were important. We also found that dimensions of access to care were interdependent, with one dimension of access to antenatal care having the potential to promote or disrupt another dimension of access.

## Discussion

This qualitative evidence synthesis and evidence-based conceptual model demonstrated that initial and ongoing access to antenatal care for ethnic minority women in European countries was highly dependent on a number of interrelated factors, including the manner in which antenatal care was provided. Women’s previous experiences of interacting with health and maternity services, the extent to which communication was facilitated, and immigration status were also key factors impacting access to antenatal care.

Our findings show that access to antenatal care for ethnic minority women in high-income European countries was mainly dependant on the nature of its provision, highlighting factors at the system level which operated to impact access to care. One particular component of antenatal care provision which was considered highly desirable by included study participants was continuity of carer. Midwifery continuity of carer is well known to foster greater support, information and satisfaction for women of all backgrounds [54, 55]. Specialist models of provision were beneficial for asylum seeking and undocumented migrant women in our included studies, but we noted a paucity of evidence on views of specialist maternity care among ethnic minority women more broadly. Policy initiatives providing targeted interventions for ethnic minority women, in the form of midwifery continuity of carer models [56], apply principles of universal proportionalism to address some of the underlying inequities faced by ethnic minority women, in an effort to reduce adverse maternal and neonatal outcomes. Yet there is a notable dearth of studies exploring ethnic minority women’s views on being ‘targeted’, and possibly therefore ‘othered’, highlighting an urgent need for research in this area. Furthermore, although some studies have suggested that continuity of carer may protect against adverse obstetric and neonatal outcomes for women with social risk factors (including ethnic minority women) [57, 58], more research is needed to understand these mechanisms fully. A further factor when considering the provision of antenatal care is that of staffing shortages experienced in some high-income European countries, such as Italy and the UK [59, 60]. An understaffed workforce can negatively impact antenatal care quality, and there have been calls for health policy focus on midwifery workforce expansion (ibid.).

This review highlights the challenges faced by women in initiating antenatal care. The promotion of the importance of early initiation of antenatal care is underexplored in high-income countries. Further research is needed to explore this in more depth, especially given the data showing ethnic minority women being more likely to delay initiation of antenatal care [61, 62].

Although care provider perspectives were not explored in this review, the contributory nature of women’s interactions with antenatal care providers in ongoing access to, and uptake of antenatal care was evident. This finding resonated with those of other studies focussing on antenatal care provider perspectives, in which cultural barriers, stereotypes and notions of deservingness were conspicuous [63, 64]. Our findings showed that provider characteristics which engendered trust, support and empowerment, and an environment where women were able to communicate and feel that their information needs were sufficiently met, were important elements supporting access to antenatal care. Negative experiences of stigmatising, discriminatory attitudes from care providers, language barriers and a failure to meet women’s basic information needs acted as explicit barriers to access. Such attitudes may be a result of maternity care providers’ unconscious racial bias including any stereotypes or beliefs that may affect their behaviours and interactions with service users and care related decision-making [65]. Supportive attributes of maternity care providers have been well recognised as a universal need for women from all backgrounds [66]. As indicated by other researchers, for ethnic minority women living within structures of former or current racial bias [67, 68], trusting and supportive relationships with care providers are especially pertinent. Inherent in such relationships is the notion of cultural safety, in which health providers recognise and reflect upon power imbalances in their care of women. The notion of cultural safety is distinct from cultural competence that focuses at an individual level on cultural knowledge which may lead to ‘othering’ [69].

Our conceptual model displays “individual-level” factors impacting ethnic minority women’s access to antenatal care. While referring to the individual-level factors, we recognise that there were wider cultural, social and political determinants which impacted the individual-level factors. As this review synthesised evidence from women’s perspectives on an individual-level, these wider determinants were not reflected in our findings as such. Nonetheless, they are worthy of discussion here. Many of the individual factors brought to light the obstacles faced by women as they navigated the processes required to initiate antenatal care and the associated health systems bureaucracy. Navigating maternity care systems was particularly challenging for women who did not speak the local language, those who were unfamiliar with local health or maternity care systems, or for asylum seeking or undocumented women who were unclear about their entitlements to maternity care and associated costs. A previous systematic review [10] acknowledged the role of social networks in sharing information about pregnancy, and explicitly identified the importance of social networks and third sector organisations in facilitating access to antenatal care for ethnic minority women in high-income countries. Nonetheless, such networks are not available for all women, necessitating interventions to provide information about, and routes to, antenatal care.

This review indicates ethnic minority women’s deeply felt need to receive information during pregnancy about how to look after themselves and their unborn baby, but this need was commonly unmet. De Freitas et al. attributed the insufficient provision of information for ethnic minority women, to care providers’ lack of time, coupled with misunderstandings of women’s needs [70]. Misunderstandings are more likely to occur when there are communication barriers, and we found communication needs cutting across all aspects of access to antenatal care for ethnic minority women who did not speak the local language. Communication barriers and the lack of suitable interpreters have been widely documented as key factors that hinder women’s ability to be involved in decision-making in their care [9, 10], which in turn lead to confusion, fear and even (re-)traumatisation [71].

Our findings, that women with precarious immigration status face obstacles to accessing antenatal care at every stage, are congruent with findings from other reviews that have focussed on migrant women [72], once again highlighting the need for antenatal care services to consider and to put in place processes to meet the specific needs of this population. Ethnic minority women’s previous negative experiences of interacting with health or maternity care services may not only deter them from accessing antenatal care, but can have wider implications for their health and wellbeing both during and after the perinatal period [73].

Our conceptual framework for access to antenatal care for ethnic minority women in high-income European countries can be effectively used to inform local, national and European regional policy and service development. We recognise that the conceptual framework requires iterative testing and refining as well as updating as new research is conducted. Nonetheless we have identified six health system-related recommendations based on this framework:

1. Interventions to promote early initiation of antenatal care and referral mechanisms for ethnic minority women need to be developed and evaluated.
2. Antenatal care services should be located geographically within the communities who they serve.
3. The provision of suitable interpreters should be viewed as an essential element of antenatal care for women who do not speak the local language sufficiently to communicate with antenatal care providers.
4. Information needs to be readily available to women with precarious immigration status regarding their legal rights and entitlements to maternity care.
5. Consideration should be given to how maternity services can identify, liaise and work with relevant local third sector organisations which play a part in facilitating access to antenatal care.
6. Antenatal care providers should receive training in cultural safety to understand the implications of care that is not trust-building, respectful and compassionate, and unconscious bias training, to address inequitable interactions with maternity care providers. While we recognise the complexities of providing culturally safe care (which are beyond the scope of this article to discuss in greater depth), we recommend that services are oriented to enable health professionals to provide culturally safe antenatal care.

## Strengths and limitations

This is the first systematic review to have developed a conceptual model for access to antenatal care for ethnic minority women in high income European countries. The framework synthesis approach that we adopted enabled us to draw on existing access to health theory. The review has certain limitations, however. Over half of the included studies focussed on new migrant women, rather than ethnic minority women who were established in the study country. This highlights the need for further research among this latter group of women as it is possible that there are variations in issues of access to antenatal care between the two groups, which may enrich or even alter our conceptual model. Including grey literature in our review may have addressed some these evidence gaps. As our review was focused on perspectives of women, we did not include perspectives of antenatal care providers although we recognise that provider perspectives are important to elucidate factors which impact access to antenatal care. We also excluded perspectives that are broadly related to maternity care in general and did not disaggregate issues of access to antenatal care from other aspects of maternity care. The majority of studies used semi-structured interviews, so the perspectives in the included studies were possibly limited to the questions that the participants were asked. For example, we noted a paucity in explorations of the impact of religious beliefs on access to care, which have been explored in studies conducted outside Europe [74, 75]. We acknowledge that using broader terms in our search strategy may have resulted in retrieval of additional papers. In line with qualitative evidence synthesis methods, our search strategy was designed to identify a diverse and rich sample of studies across settings and groups as opposed to trying to identify all the studies that existed. Whilst the sensitivity and specificity of the search strategy can be improved, we believe our approach was sufficiently comprehensive for our purpose to reach conceptual saturation [76].

## Conclusion

Our review findings showed that multiple factors affected access to antenatal care for ethnic minority women in high-income European countries. The majority of studies included in this review focused on participants who were newly arrived in the host country and further research is urgently required to explore and understand issues of access to care for ethnic minority women who were born and raised in the country in which they are accessing antenatal care. Our findings highlight the need for changes to be incorporated into antenatal care services to address barriers in accessing antenatal care in a timely fashion for ethnic minority women from the initial appointment and through the entire pregnancy, in order to contribute to an improvement in adverse obstetric and neonatal outcomes.

## Supplementary Information

**Additional file 1.**

## Data Availability

All data included in the review are publicly available research findings.

## Abbreviations

UK: United Kingdom
PRISMA: The Preferred Reporting Items for Systematic Reviews and Meta Analyses
PICOS: Population, Intervention, Comparator, Outcome, Study design
