# Supplementary material for: Ethnic minority women’s experiences of accessing antenatal care in high income European countries: a systematic review
Source: BMC Health Serv Res. 2023 Jun 10;23:612. doi: 10.1186/s12913-023-09536-y (PMC10256965; doi:10.1186/s12913-023-09536-y)
Supplement: Supplementary file 1 — Additional file 1. [file 12913_2023_9536_MOESM1_ESM.docx]

**List of high-income European countries as defined by the World Bank (2022)**

Austria

Belgium

Croatia

Czech Republic

Denmark

Estonia

Finland

France

Germany

Greece

Hungary

Ireland

Isle of Man

Italy

Latvia

Liechtenstein

Lithuania

Luxemburg

Malta

Monaco

Netherlands

Norway

Poland

Portugal

Slovak Republic

Slovenia

Spain

Sweden

Switzerland

United Kingdom
